# Anal HPV Infection in HIV-Positive Men Who Have Sex with Men from China

**DOI:** 10.1371/journal.pone.0015256

**Published:** 2010-12-06

**Authors:** Lei Gao, Feng Zhou, Xiangwei Li, Yu Yang, Yuhua Ruan, Qi Jin

**Affiliations:** 1 State Key Laboratory for Molecular Virology and Genetic Engineering, Institute of Pathogen Biology, Chinese Academy of Medical Sciences and Peking Union Medical College, Beijing, China; 2 State Key Laboratory for Infectious Disease Prevention and Control, National Center for AIDS/STD Control and Prevention, Chinese Center for Disease Control and Prevention, Beijing, China; University of Toronto, Canada

## Abstract

**Background:**

Anal HPV infection, which contributes to the development of anal warts and anal cancer, is well known to be common among men who have sex with men (MSM), especially among those HIV positives. However, HIV and anal HPV co-infection among MSM has not been addressed in China.

**Methods:**

A cross-sectional study was conducted in Beijing and Tianjin, China. Study participants were recruited using multiple methods with the collaboration of local volunteer organizations. Blood and anal swabs were collected for HIV-1 serological test and HPV genotyping.

**Results:**

A total of 602 MSM were recruited and laboratory data were available for 578 of them (96.0%). HIV and anal HPV prevalence were 8.5% and 62.1%, respectively. And 48 MSM (8.3%) were found to be co-infected. The HPV genotypes identified most frequently were HPV06 (19.6%), HPV16 (13.0%), HPV52 (8.5%) and HPV11 (7.6%). Different modes of HPV genotypes distribution were observed with respect to HIV status. A strong dose-response relationship was found between HIV seropositivity and multiplicity of HPV genotypes (p<0.001), which is consistent with the observation that anal HPV infection was an independent predictor for HIV infection.

**Conclusions:**

A high prevalence of HIV and anal HPV co-infection was observed in the MSM community in Beijing and Tianjin, China. Anal HPV infection was found to be independently associated with increased HIV seropositivity, which suggests the application of HPV vaccine might be a potential strategy to reduce the acquisition of HIV infection though controlling the prevalence of HPV.

## Introduction

Genital Human papillomaviruse (HPV) infections are very common and are sexually transmitted [1]. The outcome of HPV infection depends on its oncogenic type (low-risk or high-risk). Genital warts are most commonly associated with two low-risk types, HPV6 and HPV11 [2]. It has been well established that high-risk types, such as HPV16 and HPV18, are the primary cause of cervical cancer [3]. High-risk HPV is also suggested to play a key role for the development of anal cancer and precancerous lesions [4], [5].

In the general population, anal cancer is a rare disease. Each year it is diagnosed in about two people out of every 100,000 people [6]. However, among men who have sex with men (MSM), and especially those HIV positives, the incidence of anal cancer is significantly more prevalent and increasing annually [7]. Studies from Western countries suggested that anal HPV is present in approximately 65% of HIV negative MSM and 95% of MSM who are HIV positive [8]–[10]. The high prevalence of HPV infection is regarded to contribute to the higher risk of genital warts and anal cancer. Moreover, anal HPV infection, especially high-risk types, was suggested to be independently associated with HIV acquisition [11]–[13]. In recent years, HPV vaccines have been introduced in many countries with success to achieve significant reduction in persistent HPV infection and its related outcomes in both women and men [14]. If the causal link between HPV infection and HIV acquisition was well established, application of HPV vaccine could be a potential strategy for HIV prevention in the high-risk population.

In China, the prevalence of HIV/AIDS and other sexually transmitted diseases (STDs) is rapidly rising among MSM in the past decade, which draws great concern of the public health fields [15]. However, to our knowledge, prevalence of anal HPV infection and its association with HIV among this high-risk population has not been addressed in China. The aim of this pilot cross-sectional study was to investigate the status of HIV and anal HPV infection among MSM in Beijing and Tianjin, China.

## Results

### Subject recruitment and characteristics

In total, 607 eligible participations were interviewed and signed the informed consent, 5 of them were excluded (3 did not complete sample collection and 2 Vietnamese living in Beijing), leading to a final study population of 602 (302 from Beijing and 300 from Tianjin). As shown in Table 1, major characteristics of the study population were evaluated by site, No significant difference was found for any character (p>0.05). Therefore, subjects from the two sites were pooled together for further association analyses. The majority of participants were younger than 30 years (69.7%), Han nationality (95.0%), self-reported homosexual tendency (72.6%), and had the first homosexual act at 18 years old or later (88%). Minority nationalities were distributed in the study participants as: Hui (1.5%), Manchu (1.5%), MongoljEn (0.5%), Miao (0.3%), Uyghur (0.3%), Korean (0.3%), Chuang (0.2%), Tujia (0.2%) and Sui (0.2%). There were 23.3% participants reported a history of STDs other than HIV. Laboratory data suggested a prevalence of 8.5% of HIV seropositivity among the study population.

**Table 1:** Characteristics of the study population by study site.

	Total (N = 602)n* (%)	By site		
		Beijing (N = 302)n* (%)	Tianjin (N = 300)n* (%)	p for difference
**Age**				
≤19 years	37 (6.1)	18 (6.0)	19 (6.3)	0.195
20–29 years	383 (63.6)	184 (60.9)	199 (66.3)	
30–39 years	111 (18.4)	66 (21.9)	45 (15.0)	
≥40 years	71 (11.8)	34 (11.3)	37 (12.3)	
**Ethnicity**				
Han	570 (95.0)	285 (94.7)	285 (95.3)	0.729
Others	30 (5.0)	16 (5.3)	14 (4.7)	
**Education**				
≤9 years	108 (18.0)	64 (21.3)	44 (14.7)	0.065
10–12 years	183 (30.5)	93 (31.0)	90 (30.0)	
>12 years	309 (51.5)	143 (47.7)	166 (55.3)	
**Marriage status**				
Unmarried	467 (77.7)	232 (77.1)	235 (78.3)	0.711
Married	134 (22.3)	69 (22.9)	65 (21.7)	
**Self-reported sexual orientation**				
Homosexual	426 (72.6)	204 (69.2)	222 (76.0)	0.062
Bisexual/heterosexual	161 (27.4)	91 (30.9)	70 (24.0)	
**Ever had sex with women**				
Yes	223 (37.1)	117 (38.9)	106 (35.3)	0.370
No	378 (62.9)	184 (61.1)	194 (64.1)	
**Age at the first homosexual act**				
<18 years	72 (12.0)	41 (13.6)	31 (10.4)	0.220
≥18 years	528 (88.0)	260 (86.4)	268 (89.6)	
**Ever had STDs other than HIV-1**				
No	454 (76.7)	217 (73.6)	237 (79.8)	0.073
Yes	138 (23.3)	78 (26.4)	60 (20.2)	
**HIV-1 serostatus**				
Negative	551 (91.5)	272 (90.1)	279 (93.0)	0.196
Positive	51 (8.5)	30 (9.9)	21 (7.0)	

Abbreviation: STD, sexual transmitted disease.

*Sum may not always add up to total because of missing data.

### Anal HPV type distribution

As shown in Table 2, the genotyping results of HPV were available for 578 participants (96%), 62.1% of them were positive for at least one of targeted 26 HPV types. Among HPV positives, 47.1% (169/359) of them were multiple types infected. HIV positives were almost always infected with HPV (96.0%), and much lower prevalence was observed among HIV negatives (58.9%). HPV06 (19.6%), HPV16 (13.0%), HPV52 (8.5%) and HPV11 (7.6%) were found to be the most frequently identified types. Higher prevalence was observed for most tested types among HIV positives as compared to HIV negatives, and the difference was statistically significant for several HPV types.

**Table 2:** Anal HPV type distribution according to HIV-1 serostatus.

HPV type*	Total (N = 578)n (%)	HIV-1 serostatus		
		Negatives (N = 528)n (%)	Positives (N = 50)n (%)	p for difference
***Total***				
Any type	359 (62.1)	311 (58.9)	48 (96.0)	<0.001
Single type	190 (32.9)	169 (32.0)	21 (42.0)	0.151
Multiple types	169 (29.2)	142 (26.9)	27 (54.0)	0.002
***High-risk***				
16	75 (13.0)	58 (11.0)	17 (34.0)	<0.001
18	34 (5.9)	27 (5.1)	7 (14.0)	0.011
31	0	0	0	
33	20 (3.5)	15 (2.8)	5 (10.0)	0.008
35	2 (0.4)	2 (0.4)	0	0.663
39	36 (6.2)	34 (6.4)	2 (4.0)	0.495
45	31 (5.4)	24 (4.6)	7 (14.0)	0.005
51	21 (3.6)	15 (2.8)	6 (12)	<0.001
52	49 (8.5)	40 (7.6)	9 (18.0)	0.011
26/55	16 (2.8)	14 (2.7)	2 (4.0)	0.579
56	6 (1.0)	4 (0.8)	2 (4.0)	0.031
58	38 (6.6)	32 (6.1)	6 (12.0)	0.105
59	22 (3.8)	22 (4.2)	0	0.141
66	2 (0.4)	0	2 (4.0)	<0.001
68	24 (4.2)	24 (4.5)	0	0.124
83	12 (2.1)	11 (2.1)	1 (2.0)	0.969
***Low-risk***				
6	113 (19.6)	98 (18.6)	15 (30.0)	0.051
11	44 (7.6)	39 (7.4)	5 (10.0)	0.503
40/42/44	10 (1.7)	8 (1.5)	2 (4.0)	0.197
61/73	45 (7.8)	39 (7.4)	6 (12.0)	0.243

*Classification of HPV types, according to carcinogenicity, is recommended by Tellgenplex™ HPV DNA Test.

### Risk factors for anal HPV infection

The association between potential risk factors and anal HPV infection was assessed by logistic regression analysis as shown in Table S1 and Table S2. Univariate analyses suggested minority nationality, taking oral sex and anilinction as regular sexual behaviors, and ever found sexual partner in gay venues (e.g., MSM clubs, bars, parks and bathhouses) were associated with increased risk of anal HPV infection. Lower prevalence was observed for those with higher education level (>12 years). Then, such identified significant predictors, together with age, were included in a multiple logistic regression analysis. The associations of education and oral sex were not significant anymore. Stepwise backward selection identified minority nationality, oral sex, and ever found sexual partner in gay venues were independent predictors with adjusted OR of 3.28 (1.21–8.89), 1.76 (1.22–2.55), and 1.86 (1.25–2.76) respectively.

### Risk factors for HIV infection

As shown in Table S3 and Table S4, ethnicity, education, age at the first homosexual act, frequency of homosexual behaviors in the past 6 months, ever found sexual partners in gay venues and anal HPV status were significantly associated with HIV seropositivity in univariate analyses. Only the associations for the latter two predictors were still significant in the multiple logistic regression analysis. Stepwise backward selection also identified these two predictors, ever found sexual partners in gay venues and anal HPV status, were independent risk factors for HIV seropositivity with adjusted OR of 3.76 (1.90–7.43) and 15.16 (3.62–63.60), respectively.

### The association between HIV seropositivity and anal HPV infection

The association between HIV seropositivity and anal HPV infection was further analyzed according to HPV oncogenic type and the number of co-infected HPV types as shown in Table 3. Both low-risk and high-risk type were found to be significantly related to HIV seropositivity. There was a significant association between HPV multiplicity and the prevalence of HIV.

**Table 3:** The association of anal HPV infection with HIV-1 seropositivity by oncogenic type and multiplicity of HPV types.

Anal infection of 26 HPV types	Prevalence of HIV-1 seropositivityn/N (%)	OR (95% CI)	Adjusted OR* (95% CI)
***By oncogenic type***			
Negative for any type	2/219 (0.9)	Ref.	Ref.
Only Positive for low-risk type	9/86 (10.5)	**9.35 (2.47–35.41)**	**8.62 (2.22–33.44)**
Only Positive for high-risk type	27/181 (14.9)	**14.03 (4.18–47.03)**	**11.84 (3.47–40.48)**
Positive for both	12/92 (13.0)	**12.00 (3.30–43.60)**	**9.90 (2.67–36.73)**
***By multiplicity of HPV types***			
0	2/219 (0.9)	Ref.	Ref.
1	21/190 (11.1)	**9.94 (2.92–33.86)**	**8.71 (2.51–30.27)**
2	17/115 (14.8)	**13.88 (3.98–48.42)**	**11.54 (3.25–40.98)**
3	5/32 (15.6)	**14.82 (3.35–65.45)**	**14.44 (3.15–66.13)**
≥4	5/22 (22.7)	**23.53 (5.18–106.89)**	**16.12 (3.33–77.99)**
p for trend		**<0.001**	**<0.001**

Abbreviation: CI, confidence intervals; OR, odds ratio.

*Adjusted for age, ethnicity, education, age at the first homosexual act, frequency of homosexual behaviors in the past 6 months and ever found sexual partners in gay venues.

## Discussion

This pilot study, for the first time, investigated molecular epidemiological characteristics of anal HPV infection among MSM in China. In addition, association between HIV seropositivity and anal HPV infection was also addressed. In a total of 602 study subjects, HIV and anal HPV prevalence were 8.5% and 62.1%, respectively. And 96.0% HIV positives were anal HPV co-infected. HPV infection was identified as an independent predictor for HIV seropositivity, and a significant relationship was found between HIV seropositivity and multiplicity of HPV types.

In the past decades, several studies have shown that anal HPV infection is common among MSM and is almost always present in HIV positive MSM. In 1998, a study from the United States reported 93% of 346 HIV positive and 61% of 262 HIV negative MSM had anal HPV infection [8]. In recent years, several studies from different MSM populations consistently reported such a universal prevalence of anal HPV infection and multiple HPV type infection was found to be common [16]–[19]. Moreover, anal HPV infection, especially high-risk type, was suggested to be independently associated with HIV acquisition [11]–[13]. The mechanisms are not clear yet, but there is a biology plausibility that HPV infection related lesions may lead to an active cell-mediated immune response through recruitment of macrophages and T lymphocytes which are HIV-susceptible cells and may facilitate HIV acquisition [20], [21]. In addition, oncogenic HPV and the presence of multiple HPV genotypes are correlated with the presence of high-grade lesions which could be the risky lesions for HIV acquisition [22]. What's more, a direct interaction between HPV and HIV was also suggested to contribute to this association [23], [24]. Whatever, vaccination based control of anal HPV infection will not only prevent HPV related diseases but also regarded to be a promising tool for prevention of HIV infection [11], [13], [17]. In China, MSM is a high-risk population for STDs including HIV infection [25]. It is necessary to pay attention to this population with respect to anal HPV infection and its related diseases.

High prevalence of HIV and anal HPV infection was observed in our study subjects, which is consistent with the reports from Western countries. HPV06, HPV16, HPV52, and HPV11 were found to be most frequently identified types among study population. But a different type distribution was observed by HIV status, the prevalence of several HPV types was significantly higher among HIV positives than among negatives. A potential underlying mechanism might be the association between HIV acquisition and HPV infection was type dependent. Recently, Auvert and his colleagues reported that high-risk HPV, but not low-risk HPV, was significantly associated with HIV incidence [11]. However, the effect of low-risk HPV was assessed after controlling for the status of high-risk HPV. It is not clear whether the authors considered the influence of those who were high-risk and low-risk HPV co-infected. In our study, the subjects were grouped into three subgroups according to HPV type (as shown in Table 3): positive only for high-risk HPV, positive only for low-risk HPV, and positive for both. Our results suggested both low-risk and high-risk HPV were associated with increased HIV seropositivity, and the association for the latter one was moderately higher. Besides varied strength of the association for different HPV type, a possible effect of HIV infection on the natural history of HPV could not be excluded. It is suggested that cell-mediated immune response to HPV is lacking as a result of HIV infection and contributes to reactivation of a latent HPV infection [24]. Further studies are warranted to clarify the underlying mechanisms for the different HPV type distribution between HIV negatives and positives.

Actually, there is a debate on the direction of the causal link between HIV acquisition and HPV infection. Several studies have focused on the effects of HIV infection on HPV prevalence and suggested that HIV infection induced immune depression increased the risk of HPV infection [26]–[30]. Despite its biology plausibility, our present study observed a significant relationship between HIV seropositivity and multiplicity of HPV types, which is consistent with the report by Chin-Hong and colleagues [12]. This finding provides a further evidence to support the causality link suggested by prospective studies [12], [13]. However, we can not exclude a possibility that the relation is true in both directions due to the limitation of our study design. The complicated interaction between HPV and HIV and its impact on the development of clinical outcomes should be further investigated. To evaluate the protective effect of HPV vaccine against HIV acquisition would be a reasonable and valuable way to explore the underlying key mechanism [31].

In the interpretation of our data, some limitations have to be considered. First, data of socio-demographic and risk behaviors were based on an interview based standardized questionnaire. Potential bias caused by inaccurate response can therefore not be excluded. Second, our study participants might not represent the general MSM population in the two sites due to the potential limitation of enrollment methods. Potential selection bias should be kept in mind when interpret the prevalence data. Third, it should be kept in mind that HPV infected cells might not be collected successfully using the current sampling method, and accuracy of the test results might also be influenced by the quality of the samples. Fourth, cross-sectional study design has its limitation on association analysis. Nevertheless, our results provided evidence to the findings of studies from countries other than China [11]–[13].

In conclusion, high prevalence of HIV seropositivity and anal HPV infection was observed among MSM from Beijing and Tianjin, China. HPV infection was identified as an independent predictor for HIV infection. Different distribution of HPV type by HIV status suggested a HPV type dependent interaction. If the causal link between HPV and HIV infection was confirmed by further studies, it would be a potential strategy to reduce the acquisition of HIV infection through application of HPV vaccine in the MSM population.

## Materials and Methods

### Ethic statement

The study was approved by the Ethics Committees of the Institute of Pathogen Biology, Chinese Academy of Medical Sciences & Peking Union Medical College. Written informed consent was obtained from each study participant before the interview and testing.

### Study population

Study participants were recruited from sites in 2 cities (Beijing and Tianjin), between March and July 2010, though local non-government organizations (Beijing Rainbow Volunteers Workstation and Tianjin Deep Blue Volunteers Workgroup). Multiple methods were used for recruitment including website advertisement, distributing flyers with study-related information at MSM-frequented venues (e.g., MSM clubs, bars, parks and bathhouses), and eligible study participants were also encouraged to refer their peers to attend the study. Those eligible to participate were males, at least 18 years old, ever had homosexual behaviors, willing to take tests for HIV/anal HPV infection and syphilis, and physically able and willing to provide written informed consent. Those study participants who were tested positive for HIV or any susceptible symptoms for STDs were informed by study personnel confidentially and referred to treatment at the Institute of STD/AIDS Prevention and Treatment, Xicheng District Center for Disease Prevention and Control, Beijing and the STD/AIDS clinic at affiliated hospital of Tianjin Medical University.

### Data collection

Data were collected using a questionnaire administered by a trained interviewer in a private room. Each study participant was assigned a unique code that was used to link the questionnaire and specimens. Data were collected, including self-reported socio-demographic characteristics (e.g., age, income, ethnicity, education, employment, and marriage status), sexual behaviors in the past 6 months, knowledge on STDs, history of STDs, and lifestyle factors.

### Sample collection and laboratory tests

Blood samples were collected for test of HIV serology. The HIV infection status was determined by an enzyme immunoassay (Wantai Biological Medicine Company, Beijing, China). And positive tests were confirmed by HIV-1/2 Western blot assay (HIV Blot 2.2 WB; Genelabs Diagnostics, Singapore).

Trained personnel at each site collected anal samples by rotating a saline water moistened nylon flocked swab in the anal canal for about 2 minutes. The swab was then kept in 3 mL of sample transport medium for Tellgenplex™ HPV DNA Test (Tellgen Life Science, Shanghai, China). Tellgenplex™ HPV DNA Test is based on suspension bead array method to identify HPV types. Suspension bead array is a technical based on PCR,beads coated hybridization, flow cytometry, lasers and digital signal processing. Target HPV DNA-specific probes can be coated on the surface of spectrally addressable polystyrene beads. As the beads are internally labeled with a ratio of two spectrally distinct fluorophores, the bead lots are assignable to class-specific HPV subtypes. Mixtures of different bead suspensions can be used in the same well of a 96-well plate format allowing for multiplex analysis. Tellgenplex™ HPV DNA Test provides identification of 19 recommended high-risk subtypes (16, 18, 26, 31, 33, 35, 39, 45, 51, 52, 53, 55, 56, 58, 59, 66, 68, 82, and 83) which are associated with cervical caner. Beside that, the kit also provides detection of 7 low-risk subtypes (6, 11, 40, 42, 44, 61, and 73). In order to minimize the possible nonspecific competition between partial probes, the manufacture suggests to report following types combined: 26/55, 40/42/44, and 61/73.

### Statistical analysis

Questionnaires were double entered and compared with EpiData software (EpiData 3.02 for Windows, The Epi Data Association Odense, Denmark). After cleaning, the data were then converted and analyzed using Statistical Analysis System (SAS 9.1 for Windows; SAS Institute Inc., NC, USA).

Study population was characterized by site with respect to age, ethnicity, education, marriage status, self-reported sexual orientation, ever had sex with women, age at the first homosexual act, is anal sex a regular sex behavior, ever had STDs other than HIV, and current status of HIV. Differences between sites in these variables were assessed with Pearson chi-square test.

The associations of potential risk factors with HIV/HPV infection were estimated by means of odds ratios (OR) and 95% confidence intervals (95% CI) using unconditional logistic regression. Age and other covariates with a significant association in univariate analyses were included in a further multiple logistic regression analysis. Stepwise backward multiple logistic regression analysis was then used to identify those predictors that were independently associated with HIV/HPV infection. The significance level for the predictors to stay in the model was 0.05. In order to investigate the combined effect of HPV types, the risk of HIV seropositivity was then assessed according to the number of HPV types in each sample. Tests for trend by were performed by treating categories of the number as continuous variable in the logistic regression analysis.

## Supporting Information

Table S1 Risk factors associated with anal infection for 26 HPV subtypes (part 1/2).
(DOC)

Table S2 Risk factors associated with anal infection for 26 HPV subtypes (part 2/2).
(DOC)

Table S3 Risk factors associated with prevalence of HIV-1 seropositivity (part 1/2).
(DOC)

Table S4 Risk factors associated with prevalence of HIV-1 seropositivity (part 2/2).
(DOC)
