# Correction: Anal HPV Infection in HIV-Positive Men Who Have Sex with Men from China

**DOI:** 10.1371/annotation/322ccfc2-9407-4c6d-b08f-0ba8ffe8918c

**Published:** 2011-01-07

**Authors:** Lei Gao, Feng Zhou, Xiangwei Li, Yu Yang, Yuhua Ruan, Qi Jin

Assignment of Equal Contributions was inadvertently omitted. Dr. Lei Gao and Dr. Feng Zhou contributed equally to this paper. 

