# Supplementary material for: Anal HPV Infection in HIV-Positive Men Who Have Sex with Men from China
Source: PLoS One. 2010 Dec 6;5(12):e15256. doi: 10.1371/journal.pone.0015256 (PMC2997781; doi:10.1371/journal.pone.0015256)
Supplement: Table S1 — Risk factors associated with anal infection for 26 HPV subtypes (part 1/2). (DOC) [file pone.0015256.s001.doc]

**Table S1. Risk factors associated with anal infection for 26 HPV subtypes (part 1/2)**

| **Variables** | **Prevalence**  **n/N* (%)** | **OR (95% CI)** | **Adjusted OR (95% CI) ^#^** |
| --- | --- | --- | --- |
| **Age** | | | |
| ≤19 years | 24/34 (70.6) | Ref. | Ref. |
| 20-59 years | 229/369 (62.1) | 0.68 (0.32-1.47) | 0.80 (0.32-1.47) |
| 30-39 years | 69/107 (64.5) | 0.76 (0.33-1.75) | 0.73 (0.30-1.74) |
| ≥40 years | 37/68 (54.4) | 0.50 (0.21-1.20) | 0.46 (0.19-1.16) |
| **Ethnicity** | | | |
| Han | 332/546 (60.8) | Ref. | Ref. |
| Others | 25/30 (83.3) | **3.22 (1.22-8.55)** | **3.20 (1.17-8.70)** |
| **Education** | | | |
| ≤9 years | 75/107 (70.1) | Ref. | Ref. |
| 10-12 years | 109/172 (63.4) | 0.72 (0.43-1.21) | 0.82 (0.48-1.37) |
| >12 years | 173/297 (58.3) | **0.58 (0.36-0.93)** | 0.65 (0.39-1.08) |
| **Marriage status** | | | |
| Unmarried | 276/446 (61.9) | Ref. |  |
| Married | 82/131 (62.6) | 1.03 (0.69-1.54) |  |
| **Self-reported sexual orientation** | | | |
| Homosexual | 256/406 (63.1) | Ref. |  |
| Bisexual/heterosexual | 98/158 (62.0) | 0.96 (0.66-1.40) |  |
| **Ever had sex with women** | | | |
| Yes | 136/218 (62.4) | Ref. |  |
| No | 223/360 (61.9) | 0.98 (0.69-1.39) |  |
| **Age at the first homosexual act** | | | |
| <18 years | 47/68 (69.1) | Ref. |  |
| ≥18 years | 310/508 (61.0) | 0.70 (0.40-1.21) |  |
| **Ever had other STDs** | | | |
| No | 266/436 (61.0) | Ref. |  |
| Yes | 84/132 (63.6) | 1.12 (0.75-1.67) |  |
| **Is anal sex a regular homosexual behavior?** | | | |
| No | 88/131 (67.2) | Ref. |  |
| Yes | 270/446 (60.5) | 0.75 (0.50-1.13) |  |
| **Is oral sex a regular** **homosexual behavior?** | | | |
| No | 86/161 (53.4) | Ref. | Ref. |
| Yes | 272/416 (65.4) | **1.65 (1.14-2.38)** | 1.15 (0.76-1.74) |
| **Is anilinction a regular homosexual behavior?** | | | |
| No | 229/400 (57.3) | Ref. | Ref. |
| Yes | 129/177 (72.9) | **2.01 (1.36-2.95)** | **1.89 (1.23-2.91)** |

Abbreviation: CI, confidence intervals; OR, odds ratio; STD, sexual transmitted disease.

^*^ Sum may not always add up to total because of missing data.

^#^ Adjusted for age and covariant which were significantly associated with HIV-1 seropositivity in the univariate analysis (e.g., ethnicity, education, oral sex as a regular homosexual behavior, anilinction as a regular sex behavior, ever found sexual partners in gay venues).
