# Supplementary material for: Anal HPV Infection in HIV-Positive Men Who Have Sex with Men from China
Source: PLoS One. 2010 Dec 6;5(12):e15256. doi: 10.1371/journal.pone.0015256 (PMC2997781; doi:10.1371/journal.pone.0015256)
Supplement: Table S2 — Risk factors associated with anal infection for 26 HPV subtypes (part 2/2). (DOC) [file pone.0015256.s002.doc]

**Table S2. Risk factors associated with anal infection for 26 HPV subtypes (part 2/2)**

| **Variables** | **Prevalence**  **n/N* (%)** | **OR (95% CI)** | **Adjusted OR (95% CI) ^#^** |
| --- | --- | --- | --- |
| **Frequency of homosexual behaviors in the past 6 months** | | | |
| <once a week | 185/303 (61.1) | Ref. |  |
| ≥once a week | 172/272 (63.2) | 1.10 (0.78-1.54) |  |
| **Condom use during insertive anal sex in the past 6 months** | | | |
| Always | 132/214 (61.7) | Ref. |  |
| Sometimes/Never | 70/128 (61.7) | 1.00 (0.64-1.57) |  |
| **Condom use during receptive anal sex in the past 6 months** | | | |
| Always | 141/214 (65.9) | Ref. |  |
| Sometimes/Never | 95/151 (62.9) | 0.88 (0.57-1.36) |  |
| **Condom use during oral sex in the past 6 months** | | | |
| Always | 34/45 (75.6) | Ref. |  |
| Sometimes/Never | 283/454 (62.3) | 0.54 (0.26-1.09) |  |
| **Number of homosexual partners ever had** | | | |
| ≤10 | 172/291 (59.1) | Ref. |  |
| 10-50 | 132/200 (66.0) | 1.34 (0.92-1.95) |  |
| >50 | 54/86 (62.8) | 1.17 (0.71-1.92) |  |
| **Ever found sexual partners in gay venues** | | | |
| No | 188/332 (56.6) | Ref. | Ref. |
| Yes | 170/245 (69.4) | **1.74 (1.23-2.46)** | **1.52 (1.02-2.27)** |
| **Ever had multiple partner sex in the past year** | | | |
| No | 319/516 (61.2) | Ref. |  |
| Yes | 37/58 (63.8) | 1.09 (0.62-1.92) |  |
| **Ever had paid sex with man in the past year** | | | |
| No | 320/520 (61.5) | Ref. |  |
| Yes | 33/48 (68.8) | 1.38 (0.73-2.60) |  |

Abbreviation: CI, confidence intervals; OR, odds ratio; STD, sexual transmitted disease.

^*^ Sum may not always add up to total because of missing data.

^#^ Adjusted for age and covariant which were significantly associated with HIV-1 seropositivity in the univariate analysis (e.g., ethnicity, education, oral sex as a regular homosexual behavior, anilinction as a regular sex behavior, ever found sexual partners in gay venues).
