# Supplementary material for: Anal HPV Infection in HIV-Positive Men Who Have Sex with Men from China
Source: PLoS One. 2010 Dec 6;5(12):e15256. doi: 10.1371/journal.pone.0015256 (PMC2997781; doi:10.1371/journal.pone.0015256)
Supplement: Table S3 — Risk factors associated with prevalence of HIV-1 seropositivity (part 1/2). (DOC) [file pone.0015256.s003.doc]

**Table S3. Risk factors associated with prevalence of HIV-1 seropositivity (part 1/2)**

| **Variables** | **Prevalence**  **n/N* (%)** | **OR (95% CI)** | **Adjusted OR^#^ (95% CI)** |
| --- | --- | --- | --- |
| **Age** | | | |
| ≤19 years | 3/37 (8.1) | Ref. | Ref. |
| 20-59 years | 31/383 (8.1) | 1.00 (0.29-3.44) | 1.33 (0.35-5.08) |
| 30-39 years | 10/111 (9.0) | 1.12 (0.29-4.32) | 1.23 (0.29-5.29) |
| ≥40 years | 7/71 (9.9) | 1.24 (0.30-5.10) | 1.48 (0.32-6.81) |
| **Ethnicity** | | | |
| Han | 45/570 (7.9) | Ref. | Ref. |
| Others | 6/30 (20.0) | **2.92 (1.13-7.50)** | 2.34 (0.84-6.54) |
| **Education** | | | |
| ≤9 years | 15/108 (13.9) | Ref. | Ref. |
| 10-12 years | 19/183 (10.4) | 0.73 (0.36-1.51) | 1.00 (0.45-2.18) |
| >12 years | 17/309 (5.5) | **0.37 (0.18-0.77)** | 0.79 (0.35-1.78) |
| **Marriage status** | | | |
| Unmarried | 41/467 (8.8) | Ref. |  |
| Married | 10/134 (7.5) | 0.84 (0.41-1.72) |  |
| **Self-reported sexual orientation** | | | |
| Homosexual | 40/426 (9.4) | Ref. |  |
| Bisexual/heterosexual | 11/161 (6.8) | 0.70 (0.35-1.42) |  |
| **Ever had sex with women** | | | |
| Yes | 18/223 (8.1) | Ref. |  |
| No | 33/378 (8.7) | 1.09 (0.60-1.98) |  |
| **Age at the first homosexual act** | | | |
| <18 years | 11/72 (15.3) | Ref. | Ref. |
| ≥18 years | 40/528 (7.6) | **0.46 (0.22-0.93)** | 0.55 (0.25-1.21) |
| **Ever had other STDs** | | | |
| No | 36/454 (7.9) | Ref. |  |
| Yes | 14/138 (10.1) | 1.31 (0.69-2.51) |  |
| **Is anal sex a regular homosexual behavior?** | | | |
| No | 11/135 (8.2) | Ref. |  |
| Yes | 40/466 (8.6) | 1.06 (0.53-2.12) |  |
| **Is oral sex a regular homosexual behavior?** | | | |
| No | 11/168 (6.6) | Ref. |  |
| Yes | 40/433 (9.2) | 1.45 (0.73-2.90) |  |
| **Is anilinction a regular homosexual behavior?** | | | |
| No | 35/417 (8.4) | Ref. |  |
| Yes | 16/184 (8.7) | 1.04 (0.56-1.93) |  |

Abbreviation: CI, confidence intervals; OR, odds ratio; STD, sexual transmitted disease.

^*^ Sum may not always add up to total because of missing data.

^#^ Adjusted for age and covariant which were significantly associated with HIV-1 seropositivity in the univariate analysis (e.g., ethnicity, education, age at the first homosexual act, frequency of homosexual behaviors in the past 6 months, ever found sexual partners in gay venues and anal HPV infection).
