# Supplementary material for: Anal HPV Infection in HIV-Positive Men Who Have Sex with Men from China
Source: PLoS One. 2010 Dec 6;5(12):e15256. doi: 10.1371/journal.pone.0015256 (PMC2997781; doi:10.1371/journal.pone.0015256)
Supplement: Table S4 — Risk factors associated with prevalence of HIV-1 seropositivity (part 2/2). (DOC) [file pone.0015256.s004.doc]

**Table S4. Risk factors associated with prevalence of HIV-1 seropositivity (part 2/2)**

| **Variables** | **Prevalence**  **n/N* (%)** | **OR (95% CI)** | **Adjusted OR^#^ (95% CI)** |
| --- | --- | --- | --- |
| **Frequency of homosexual behaviors in the past 6 months** | | | |
| <once a week | 19/315 (6.0) | Ref. | Ref. |
| ≥once a week | 32/284 (11.3) | **1.98 (1.09-3.58)** | 1.64 (0.86-3.11) |
| **Condom use during insertive anal sex in the past 6 months** | | | |
| Always | 16/224 (7.1) | Ref. |  |
| Sometimes/Never | 8/132 (6.1) | 0.84 (0.35-2.02) |  |
| **Condom use during receptive anal sex in the past 6 months** | | | |
| Always | 23/223 (10.3) | Ref. |  |
| Sometimes/Never | 15/159 (9.4) | 0.91 (0.46-1.80) |  |
| **Condom use during oral sex in the past 6 months** | | | |
| Always | 5/45 (11.1) | Ref. |  |
| Sometimes/Never | 38/475 (8.0) | 0.70 (0.26-1.87) |  |
| **Number of homosexual partners ever had** | | | |
| ≤10 | 21/299 (7.0) | Ref. |  |
| 10-50 | 18/212 (8.5) | 1.23 (0.64-2.37) |  |
| >50 | 12/90 (13.3) | 2.04 (0.96-4.32) |  |
| **Ever found sexual partners in gay venues** | | | |
| No | 13/346 (3.8) | Ref. | Ref. |
| Yes | 38/255 (14.9) | **4.48 (2.34-8.61)** | **3.29 (1.60-6.75)** |
| **Ever had multiple partner sex in the past year?** | | | |
| No | 44/537 (8.2) | Ref. |  |
| Yes | 7/61 (11.5) | 1.45 (0.62-3.38) |  |
| **Ever had paid sex with man in the past year?** | | | |
| No | 44/541 (8.1) | Ref. |  |
| Yes | 7/51 (13.7) | 1.80 (0.76-4.23) |  |
| **Anal HPV infection (any type)** | | | |
| Negative | 2/219 (0.9) | Ref. | Ref. |
| Positive | 48/359 (13.4) | **16.75 (4.03-69.63)** | **14.31 (3.40-60.17)** |

Abbreviation: CI, confidence intervals; OR, odds ratio; STD, sexual transmitted disease.

^*^ Sum may not always add up to total because of missing data.

^#^ Adjusted for age and covariant which were significantly associated with HIV-1 seropositivity in the univariate analysis (e.g., ethnicity, education, age at the first homosexual act, frequency of homosexual behaviors in the past 6 months, ever found sexual partners in gay venues and anal HPV infection).
